# Traffic-related air pollution, biomarkers of metabolic dysfunction, oxidative stress, and CC16 in children

**DOI:** 10.1038/s41370-021-00378-6

**Published:** 2021-08-20

**Authors:** Amy L. Zhang, John R. Balmes, Liza Lutzker, Jennifer K. Mann, Helene G. Margolis, Tim Tyner, Nina Holland, Elizabeth M. Noth, Fred Lurmann, S. Katharine Hammond, Stephanie M. Holm

**Affiliations:** 1 Division of Environmental Health Sciences, School of Public Health, University of California Berkeley, Berkeley, CA USA; 2 Western States Pediatric Environmental Health Specialty Unit, San Francisco, CA USA; 3 Department of Medicine, University of California, San Francisco, San Francisco, CA USA; 4 Department of Internal Medicine, University of California, Davis, Davis, CA USA; 5 University of California, San Francisco-Fresno, Fresno, CA USA; 6 Central California Asthma Collaborative, Fresno, USA; 7 Sonoma Technology, Petaluma, CA USA

**Keywords:** Traffic-related Air Pollution, Early Life Childhood Exposure, Oxidative Stress, Metabolic Dysregulation, Polycyclic Aromatic Hydrocarbons, Low-SES Populations

## Abstract

**Background:**

Previous research has revealed links between air pollution exposure and metabolic syndrome in adults; however, these associations are less explored in children.

**Objective:**

This study aims to investigate the association between traffic-related air pollutants (TRAP) and biomarkers of metabolic dysregulation, oxidative stress, and lung epithelial damage in children.

**Methods:**

We conducted cross-sectional analyses in a sample of predominantly Latinx, low-income children (*n* = 218) to examine associations between air pollutants (nitrogen dioxide (NO_2_), nitrogen oxides (NO_*x*_), elemental carbon, polycyclic aromatic hydrocarbons, carbon monoxide (CO), fine particulates (PM_2.5_)) and biomarkers of metabolic function (high-density lipoprotein (HDL), hemoglobin A1c (HbA1c), oxidative stress (8-isoprostane), and lung epithelial damage (club cell protein 16 (CC16)).

**Results:**

HDL cholesterol showed an inverse association with NO_2_ and NO_*x*_, with the strongest relationship between HDL and 3-month exposure to NO_2_ (–15.4 mg/dL per IQR increase in 3-month NO_2_, 95% CI = –27.4, –3.4). 8-isoprostane showed a consistent pattern of increasing values with 1-day and 1-week exposure across all pollutants. Non-significant increases in % HbA1c were found during 1-month time frames and decreasing CC16 in 3-month exposure time frames.

**Conclusion:**

Our results suggest that TRAP is significantly associated with decreased HDL cholesterol in longer-term time frames and elevated 8-isoprostane in shorter-term time frames. TRAP could have the potential to influence lifelong metabolic patterns, through metabolic effects in childhood.

## Introduction

Growing evidence suggests a link between PM_2.5_ exposure and metabolic dysfunction at a population level [1]. Metabolic syndrome and its components, such as insulin resistance, central adiposity, elevated blood pressure, and dyslipidemia [2], have all been shown to have a positive association with air pollution exposure [3–7]. This is thought to be due to higher levels of oxidative stress [8–10] and upregulated inflammatory responses in tissues of distant organs, such as the liver, pancreas, and adipose tissue [4, 6]. These processes can lead to clinically harmful effects such as glucose intolerance related to insulin resistance and increased cardiovascular morbidity [6, 11–14].

While the relationship between traffic-related air pollution (TRAP) and effects on metabolic dysregulation is well studied in adults, there are still gaps in the literature concerning these effects in children. TRAP is a category of pollutants that are emitted from motor vehicle emissions that result from fossil fuel combustion, and has been associated with adverse health effects in adulthood, such as metabolic and cardiovascular diseases [15]. Since childhood or prenatal exposures to TRAP have been hypothesized to contribute to metabolic syndrome in adults, health effects of these exposures in children have the potential to contribute to childhood disease as well as to long-term risk of adult diseases [16, 17]. Adolescent cohort studies have demonstrated significant associations between TRAP exposure and the risk factors for Diabetes Mellitus Type II, such as lower insulin sensitivity and higher abdominal adiposity, fasting insulin, and fasting glucose [6, 18, 19]. Currently, the hypothesized biological mechanism behind this association is that localized lung inflammation may trigger oxidative stress and an inflammatory response, which spills over to the circulatory system. This increases systemic inflammation, leading to adverse metabolic and cardiovascular health effects [4, 12]. More research is needed in this area to elucidate and confirm this suspected relationship, especially in pediatric cohort studies to gauge the effect of early-life exposure to traffic air pollution on later development of metabolic syndrome.

The Children’s Health and Air Pollution Study (CHAPS) is a research project focused on the adverse health effects of exposure to air pollution in childhood in Fresno, California. Located at the center of the San Joaquin Valley, Fresno residents are exposed to some of the worst air pollution in the United States [20]. Moreover, the city also has high rates of poverty and a large Hispanic/Latinx population [21]: groups that are often disproportionately affected by air pollution exposures due to close proximity to traffic sources [22]. This paper investigates the relationship between exposure to TRAP and several biomarkers of lipid and glucose metabolism (high-density lipoprotein (HDL) and hemoglobin A1c (HbA1c)), oxidative stress (urinary 8-isoprostane), and airway injury (club cell protein 16 (CC16)) in a population of low socioeconomic status, mostly Latinx children with an average age of 9.5 years. The aim is to build upon an earlier CHAPS analysis that assessed data from the cohort at age 7 and found significant associations between longer-term exposures between TRAP and HbA1c and systolic blood pressure, as well as shorter-term exposures between TRAP and urinary 8-isoprostane [23]. This follow-up cross-sectional analysis is focused on biomarker data from visits 2 years after that baseline visit, including new biomarkers not measured previously (HDL and CC16). We hypothesized that we would see similar patterns related to TRAP exposure in these biomarkers as was seen in the prior analyses (increases in HbA1c and systolic blood pressure), with decreases in HDL and changes in CC16 that could be time-frame dependent (increases in the short-term with decreases associated with longer-term exposures).

## Methods

### Study population

The data for these analyses were collected during the CHAPS, a prospective cohort study assessing the impact of air pollution on the health of children living in the Fresno metropolitan area. This study originally recruited 6- to 8-year-old children from elementary schools in Fresno during 2015–2017. Of the 299 children initially recruited into the cohort, 73% were retained and had a visit approximately 2 years later, which resulted in the 8- to 10-year-old study population for this project (Supplementary Fig. 1). The details of the recruitment process are presented in a prior publication [23]. A subset of the CHAPS participants (*n* = 122) had an additional biomarker (CC16) assessed when additional funding became available.

At the follow-up study visit, each child participant’s parent or guardian was interviewed using a detailed, structured health and general history questionnaire, and for each child participant, a non-fasting blood sample and urine sample were obtained. The questionnaire was offered to participants’ parents or guardians in either English or Spanish and assessed participant demographics, including sex, age, and race/ethnicity, in addition to parental socioeconomic indicators such as annual household income, parental education levels, parental employment, and home ownership. Standing height was measured with a stadiometer and weight with a digital scale; from these BMI was calculated to use in describing the cohort [24]. All study protocols were approved by the Institutional Review Boards at the University of California, Berkeley and Stanford University.

### Outcome measurement

Blood specimens were collected by venipuncture by a trained phlebotomist, with serum collected in serum separator tubes and whole blood collected in EDTA vacutainers (Becton, Dickinson and Company, Franklin Lakes, NJ). The samples for HDL (measured in mg/dL) and % HbA1c measurement were retrieved at room temperature within 24 h of draw and assayed by a commercial laboratory (LabCorp) using standard clinical laboratory techniques. In order to minimize participant burden and maximize study participation, the study’s selection of biomarkers did not require children to fast before the visit and blood draws. Urine collected to assay 8-isoprostane, CC16, and creatinine was shipped overnight on a gel pack within 24 h, or frozen before shipping to the Holland laboratory at UC Berkeley for urine analysis.

CC16 was assayed for a subset of participants at the same time as the CHAPS 9-year-old visit. CC16 was determined in urine by a commercially available ELISA kit (IBL-America, Minneapolis, MN). Samples were analyzed in duplicate, according to the manufacturer’s protocol, and additional quality controls included random repeats and lab controls. The limit of detection (LOD) for the CC16 assay was 2 ng/mL. The variability in readings (coefficient of variation) was 6.5% for duplicates and the random repeats were also within 10%. Creatinine concentrations were determined in urine using commercially available ELISA (Oxford Biomedical Research, MI). Samples were randomized across plates and the coefficient of variation for creatinine was less than 3%. There is debate about whether CC16 measurements should be adjusted for creatinine [25], thus a CC16/creatinine ratio was also calculated for use in a sensitivity analysis.

Urinary total 8-isoprostane was measured in the banked samples using an ELISA kit (Oxford Biomedical Research, Rochester Hills, MI) as previously described [26]. Briefly, urine samples were pre-treated with beta-glucuronidase (Oxford Biomedical Research, Rochester Hills, MI) prior to running the ELISA. The LOD for 8-isoprostane concentration was 0.08 ng/mL. Undetected oxidative stress measures were replaced with the LOD divided by the square root of 2. Additional quality assurance/quality control provisions included repeats of 5% of samples and blanks, and internal lab controls with good reproducibility of 8-isoprostane (coefficient of variation <7%). Samples were randomized across plates and the coefficient of variation for creatinine was less than 3%. All 8-isoprostane concentrations were adjusted to account for urinary dilution by dividing 8-isoprostane concentrations (ng/mL) by creatinine levels (mg/dL) with results reported in ng/mg creatinine.

### Air pollution exposure assessment

Two methods were used to model outdoor residential air pollution exposure—interpolation using inverse distance-squared weighting (for carbon monoxide (CO) and particulate matter with aerodynamic diameter of <2.5 m (PM_2.5_)) and regression modeling (for all other pollutants we considered). Complete residential address history was obtained from participating families and exposure was matched to participants’ residential street addresses. Each address was then geocoded using ESRI (Environmental Systems Research Institute) software (Redlands, CA) or Google Earth, to develop a lifetime, residential history of each participant. Individual pollutant exposures were calculated for different time periods: 1-day, the average pollutant exposure concentration in the 24 h from noon the day of biospecimen collection to noon the day prior to when the biospecimen was obtained from the participant; mean week, which is average pollutant exposure the week before study date; 1-, 3-, and 6-month averages, which are the average exposures for each of these monthly intervals prior to the study date (e.g., the 1-month average represents the average daily exposure during the month prior to the study date); and 1-year average, which is average exposure the year before study date. The major source of these pollutants in Fresno is on-road traffic, not commercial, industrial, or off-road mobile sources [27].

Linear regression with mixed effects (random and fixed) was used to develop spatiotemporal models of daily average concentrations for PAH456, elemental carbon (EC), nitrogen dioxide (NO_2_), and nitrogen oxides (NO_*x*_) incorporating data from field sampling campaigns in Fresno and Clovis [28, 29]. Briefly, hourly, quality-assured ambient pollutant (CO, NO_2_, NO_*x*_, and PM_2.5_) concentration and meteorological data collected at the local air pollution control district’s Fresno central site monitoring station (First St./Garland) and three other sites in Fresno were obtained from the U.S. Environmental Protection Agency’s Air Quality System [30]. EC and the sum of polycyclic aromatic hydrocarbons with 4, 5, and 6 rings (fluoranthene, benz[a]anthracene, chrysene, benzo[a]pyrene, benzo[b]fluoranthene, benzo[k]flouoranthene, benzo[ghi]perylene, indeno[1,2,3-cd]pyrene, and dibenz[a,h]anthracene; abbreviated PAH456) were monitored as described in a prior publication, which also discusses further details describing the CHAPS air pollution exposure assessment methods [23].

### Statistical analysis

All four biomarker outcomes were continuous variables. To quantify a relationship between biomarker levels and air pollutant exposures, regression analyses were conducted in the statistical programming language R version 4.0.4, using the packages ggplot2, gridExtra, lubridate, tidyverse, and tinytex for data manipulation/presentation, and the packages mgcv, splines, and corrarray for assessing associations between variables. Generalized additive models were used, with a p-spline smoothing function to account for seasonality. Distributions of 8-isoprostane:creatinine ratio and CC16 were right-skewed; to normalize both distributions, we conducted a log transformation of these outcome variables. Confounding variables were chosen using a directed acyclic graph (Supplementary Fig. 2) and prior knowledge [23]. All models were adjusted for the following covariates: whether the child lives with a smoker, whether the child is Latinx, physical activity, household income, and the smoothed term for the day of the study. Sensitivity analyses were performed to assess differences based on creatinine adjustment of CC16 and choice of the smoothing function for seasonality. Model results are presented for a single interquartile range (IQR) change in that pollutant, for the given exposure average (IQR values are listed in each of the results tables as well as in Supplementary Table 1).

## Results

The study cohort consisted of 218 children: 46.8% of the sample was female, and 81.7% was Latinx (Table 1). This was a sample with low socioeconomic status; 24.3% of the study participants were from a family with <$15,000 annual household income, and 70% of the study population did not own a home. Summary characteristics (median, 25th percentile, 75th percentile) for pollutant exposures are presented in Supplementary Table 1 and for outcome biomarkers in Supplementary Table 2.

**Table 1:** Socio-demographic characteristics of the CHAPS cohort (at the 9-year-old visits).

Characteristics	No. (%) or mean [SD]
Study cohort size	218
Age, mean [SD]	9.46 [0.62]
Girls (%)	102 (46.8)
*Race/ethnicity (%)*	
Latinx	178 (81.7)
African American	17 (7.8)
Non-Hispanic white	16 (7.3)
Other	7 (3.2)
Annual household income <$15K (%)	53 (24.3)
Owns home (%)	65 (29.8)
Lives with smoker (%)	42 (19.3)
*Activity level compared to children their age (%)*	
Less active	20 (9.2)
About as active	135 (61.9)
More active	63 (28.9)
Overweight^a^	42 (19.3)
Obese^a^	64 (29.4)
*Highest maternal education level (%)*	
<8th grade	28 (12.9)
Some high school	37 (17.1)
Completed high school or GED	52 (24.0)
Some college	54 (24.9)
Completed college	35 (16.1)
Advanced degree	11 (5.1)

^a^Using age- and sex-specific percentiles of the 2000 CDC growth charts, obese was defined as BMI ≥95th percentile and overweight was defined as BMI 85th to <95th percentiles.

Correlation matrices of outcome variables and exposure correlations by pollutant and exposure duration are shown in Supplementary Table 3a, b. NO_2_, PAH456, CO, and PM_2.5_ were highly correlated from 1-month through 6-month exposure averages, and NO_2_ and NO_*x*_ were very highly correlated from 1-week through 6-month exposure averages. Due to these correlations and the large number of pollutant-outcome relationships assessed, results are interpreted as the effect of TRAP, by assessing patterns in the pollutant-biomarker relationships rather than for individual pollutants presented.

**Fig. 1:**
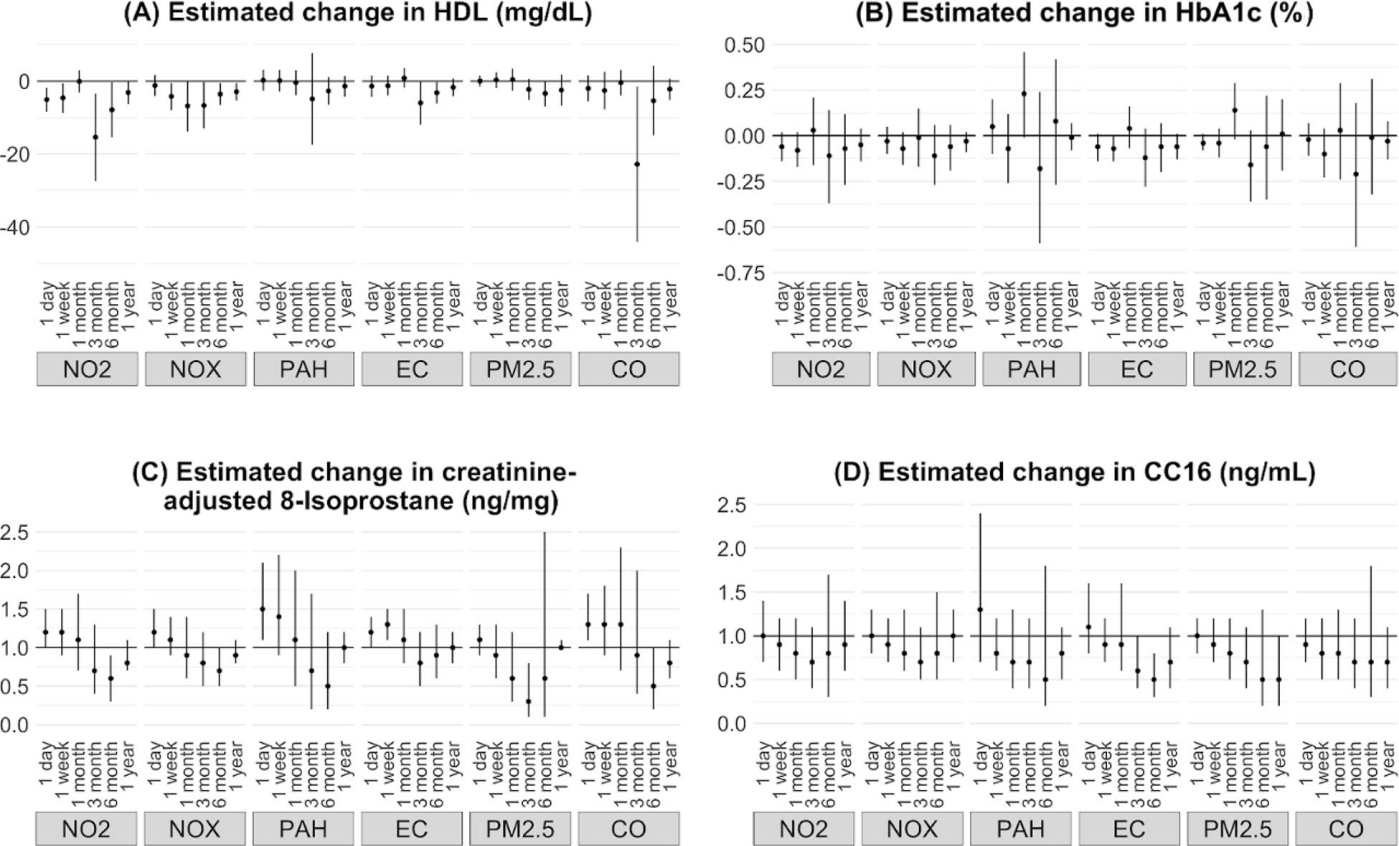
Associations of 1-day, 1-week, 1-month, 3-month, 6-month, and 1-year averages of air pollutants with estimated. **A** Change in HDL (mg/dL), **B** change in HbA1c (%), **C** multiplicative change in log_e_ creatinine-adjusted 8-isoprostane (ng/mg), and **D** multiplicative change in log_e_ CC16 (ng/mL).

For biomarkers of metabolic dysregulation, there was a consistent pattern of decreasing HDL with increasing pollutant exposure across multiple time frames (Table 2 and Fig. 1A). The largest decrease in HDL was seen in CO exposure averaged over the 3-month period (–22.8 mg/dL per 0.5 ppm increase in CO CI = –44.1, –1.53) and NO_2_ exposure averaged over a 3-month period (–15.4 mg/dL per 9.3 ppb increase in NO_2_, 95% CI = –27.4, –3.4). HDL consistently decreased in association with longer-term NO_2_ and NO_*x*_ exposure (3 months, 6 months, 1 year). Though some exposure windows did not reach statistical significance, there was also a consistent pattern of decreased HDL with increased longer-term exposure to CO, PAH456, EC, and PM_2.5_. Percent HbA1c showed a pattern of non-significant increases during 1-month exposure time frames for several pollutants (Table 3 and Fig. 1B): NO_2_, PAH456, EC, CO, and PM_2.5_.

**Table 2:** HDL cholesterol generalized additive model results.

Pollutant	1-day average	1-week average	1-month average	3-month average	6-month average	1-year average
NO_2_ (ppb) IQRs	9.4	10.1	9.7	9.3	6.0	2.2
Estimate	–5.10	–4.60	–0.10	–15.40	–7.90	–3.10
95% CI	(–8.4, –1.8)	(–8.7, –0.6)	(–3.2, 3)	(–27.4, –3.4)	(–15.4, –0.4)	(–6.3, 0.1)
*P* value	0.003	0.026	0.95	0.013	0.041	0.057
NO_*x*_ (ppb) IQRs	13.1	13.4	14.7	12.6	8.7	3.5
Estimate	–1.20	–4.20	–6.80	–6.70	–3.60	–2.90
95% CI	(–4.1, 1.7)	(–8, –0.5)	(–13.9, 0.2)	(–13, –0.3)	(–6.6, –0.5)	(–5.4, –0.5)
*P* value	0.404	0.027	0.06	0.042	0.022	0.018
PAH456 (ng/m^3^) IQRs	7.7	7.9	8.4	7.9	5.2	0.8
Estimate	0.20	0.10	–0.40	–4.90	–2.70	–1.40
95% CI	(–2.6, 3.1)	(–2.9, 3.1)	(–3.9, 3)	(–17.5, 7.7)	(–6.5, 1.1)	(–4.3, 1.4)
*P* value	0.869	0.957	0.81	0.448	0.163	0.327
EC (μg/m^3^) IQRs	0.5	0.4	0.4	0.3	0.2	0.1
Estimate	–1.40	–1.30	0.80	–6.00	–3.20	–1.70
95% CI	(–4.4, 1.5)	(–4, 1.5)	(–1.8, 3.5)	(–12, 0.1)	(–6.2, –0.3)	(–4.2, 0.7)
*P* value	0.33	0.366	0.536	0.054	0.034	0.172
CO (ppm) IQRs	0.5	0.6	0.6	0.5	0.3	0.1
Estimate	–2	–2.6	–0.4	–22.8	–5.4	–2.2
95% CI	(–5.6, 1.6)	(–7.7, 2.5)	(–4, 3.1)	(–44.1, –1.5)	(–14.9, 4.2)	(–5.2, 0.7)
*P* value	0.272	0.317	0.813	0.038	0.275	0.144
PM_2.5_ (μg/m^3^) IQRs	11.9	14.7	16.5	13.7	9.8	3.6
Estimate	0	0.2	0.8	–2.2	–1.4	–0.8
95% CI	(–0.9, 0.9)	(–1.1, 1.5)	(–1.3, 2.9)	(–7.5, 3.1)	(–3.5, 0.8)	(–3.3, 1.6)
*P* value	0.952	0.748	0.466	0.416	0.206	0.514

All results are absolute changes in HDL (mg/dL) per interquartile range (IQR) of the pollutant. These estimates come from a GAM model that adjusted for whether or not the child lives with a smoker, whether or not the child is Latinx, physical activity, household income, and a smoothed term for the day of study.

**Table 3:** HbA1c generalized additive model results.

Pollutant	1-day average	1-week average	1-month average	3-month average	6-month average	1-year average
NO_2_ (ppb) IQRs	9.4	10.1	9.7	9.3	6.0	2.2
Estimate	–0.06	–0.08	0.03	–0.11	–0.07	–0.05
95% CI	(–0.14, 0.02)	(–0.17, 0.02)	(–0.16, 0.21)	(–0.37, 0.14)	(–0.27, 0.12)	(–0.14, 0.04)
*P* value	0.12	0.12	0.78	0.39	0.46	0.31
NO_*x*_ (ppb) IQRs	13.1	13.4	14.7	12.6	8.7	3.5
Estimate	–0.03	–0.07	–0.01	–0.11	–0.06	–0.03
95% CI	(–0.1, 0.05)	(–0.16, 0.02)	(–0.17, 0.15)	(–0.27, 0.06)	(–0.19, 0.06)	(–0.09, 0.02)
*P* value	0.46	0.15	0.89	0.21	0.34	0.25
PAH456 (ng/m^3^) IQRs	7.7	7.9	8.4	7.9	5.2	0.8
Estimate	0.05	–0.07	0.23	–0.18	0.08	–0.01
95% CI	(–0.1, 0.2)	(–0.26, 0.12)	(–0.01, 0.46)	(–0.59, 0.24)	(–0.27, 0.42)	(–0.08, 0.07)
*P* value	0.5	0.48	0.06	0.4	0.66	0.88
EC (μg/m^3^) IQRs	0.5	0.4	0.4	0.3	0.2	0.1
Estimate	–0.06	–0.07	0.04	–0.12	–0.06	–0.06
95% CI	(–0.14, 0.01)	(–0.14, 0)	(–0.07, 0.16)	(–0.28, 0.04)	(–0.2, 0.07)	(–0.13, 0.01)
*P* value	0.1	0.04	0.47	0.13	0.37	0.1
CO (ppm) IQRs	0.5	0.6	0.6	0.5	0.3	0.1
Estimate	–0.02	–0.1	0.03	–0.21	–0.01	–0.03
95% CI	(–0.11, 0.07)	(–0.23, 0.04)	(–0.24, 0.29)	(–0.61, 0.18)	(–0.32, 0.31)	(–0.13, 0.08)
*P* value	0.65	0.15	0.84	0.29	0.97	0.62
PM_2.5_ (μg/m^3^) IQRs	11.9	14.7	16.5	13.7	9.8	3.6
Estimate	–0.04	–0.04	0.14	–0.16	–0.06	0.01
95% CI	(–0.08, 0.01)	(–0.12, 0.04)	(–0.02, 0.29)	(–0.36, 0.03)	(–0.35, 0.22)	(–0.19, 0.2)
*P* value	0.1	0.32	0.09	0.09	0.67	0.93

All results are absolute changes in % hemoglobin A1c per interquartile range (IQR) of the pollutant. These estimates come from a GAM model that adjusted for whether or not the child lives with a smoker, whether or not the child is Latinx, physical activity, household income, and a smoothed term for day of study.

Higher levels of 8-isoprostane were associated with short-term exposure to all measured traffic-related pollutants (Table 4 and Fig. 1C). The largest increase in 8-isoprostane was associated with 1-day lagged PAH456 exposure (1.5 times the 8-isoprostane level per 7.7 ng/m^3^ increase in PAH456, 95% CI = 1.1, 2.1) and 1-day lagged CO (1.3 times the 8-isoprostane level per 0.5 ppm increase in CO, 95% CI = 1.1, 1.7). The 8-isoprostane levels were primarily increased with short-term increases in pollutants; however, pollutant associations with 8-isoprostane dissipated at longer time frames (3 months to 1 year).

**Table 4:** 8-Isoprostane generalized additive model results.

Pollutant	1-day average	1-week average	1-month average	3-month average	6-month average	1-year average
NO_2_ (ppb) IQRs	9.4	10.1	9.7	9.3	6.0	2.2
Estimate	1.2	1.2	1.1	0.7	0.6	0.8
95% CI	(1, 1.5)	(0.9, 1.5)	(0.7, 1.7)	(0.4, 1.3)	(0.3, 0.9)	(0.7, 1.1)
*P* value	0.094	0.213	0.818	0.316	0.023	0.178
NO_*x*_ (ppb) IQRs	13.1	13.4	14.7	12.6	8.7	3.5
Estimate	1.2	1.1	0.9	0.8	0.7	0.9
95% CI	(1, 1.5)	(0.9, 1.4)	(0.6, 1.4)	(0.5, 1.2)	(0.5, 1)	(0.8, 1.1)
*P* value	0.021	0.246	0.752	0.234	0.048	0.236
PAH456 (ng/m^3^) IQRs	7.7	7.9	8.4	7.9	5.2	0.8
Estimate	1.5	1.4	1.1	0.7	0.5	1
95% CI	(1.1, 2.1)	(0.9, 2.2)	(0.5, 2)	(0.2, 1.7)	(0.2, 1.2)	(0.8, 1.2)
*P* value	0.019	0.089	0.87	0.4	0.136	0.832
EC (μg/m^3^) IQRs	0.5	0.4	0.4	0.3	0.2	0.1
Estimate	1.2	1.3	1.1	0.8	0.9	1
95% CI	(1, 1.4)	(1.1, 1.5)	(0.8, 1.5)	(0.5, 1.2)	(0.6, 1.3)	(0.8, 1.2)
*P* value	0.03	0.013	0.548	0.338	0.447	0.734
CO (ppm) IQRs	0.5	0.6	0.6	0.5	0.3	0.1
Estimate	1.3	1.3	1.3	0.9	0.5	0.8
95% CI	(1.1, 1.7)	(0.9, 1.8)	(0.7, 2.3)	(0.4, 2)	(0.2, 1)	(0.6, 1.1)
*P* value	0.014	0.143	0.434	0.826	0.059	0.228
PM_2.5_ (μg/m^3^) IQRs	11.9	14.7	16.5	13.7	9.8	6.7
Estimate	1.1	0.9	0.6	0.3	0.6	1
95% CI	(0.9, 1.3)	(0.6, 1.3)	(0.3, 1.2)	(0.1, 0.8)	(0.1, 2.5)	(1, 1.1)
*P* value	0.28	0.607	0.165	0.015	0.456	0.275

All results are multiplicative changes in 8-isoprostane to creatinine ratio (ng/mg) per interquartile range (IQR) of the pollutant. These estimates come from a GAM model that adjusted for whether or not the child lives with a smoker, whether or not the child is Latinx, physical activity, household income, and a smoothed term for the day of study.

CC16 showed a consistent pattern of decreases associated with exposures to traffic-related pollutants, even when confidence intervals cross the null (Table 5 and Fig. 1D). Across all pollutants, the 3- to 6-month exposure time frames were associated with the largest decreases in CC16 level. The association with the largest magnitude was 6-month average EC exposure, a 0.2 μg/m^3^ increase in EC was associated with 0.5 times the CC16 level (95% CI = 0.3, 0.8).

**Table 5:** CC16 generalized additive model results.

Pollutant	1-day average	1-week average	1-month average	3-month average	6-month average	1-year average
NO_2_ (ppb) IQRs	9.4	10.1	9.7	9.3	6.0	2.2
Estimate	1.00	0.90	0.80	0.70	0.80	0.90
95% CI	(0.7, 1.4)	(0.6, 1.2)	(0.5, 1.2)	(0.4, 1.1)	(0.3, 1.7)	(0.6, 1.4)
*P* value	0.98	0.35	0.36	0.14	0.49	0.64
NO_*x*_ (ppb) IQRs	13.1	13.4	14.7	12.6	8.7	3.5
Estimate	1.00	0.90	0.80	0.70	0.80	1.00
95% CI	(0.8, 1.3)	(0.7, 1.2)	(0.6, 1.3)	(0.5, 1.1)	(0.5, 1.5)	(0.7, 1.3)
*P* value	0.83	0.44	0.45	0.16	0.57	0.82
PAH456 (ng/m^3^) IQRs	7.7	7.9	8.4	7.9	5.2	0.8
Estimate	1.30	0.80	0.70	0.70	0.50	0.80
95% CI	(0.7, 2.4)	(0.6, 1.2)	(0.4, 1.3)	(0.4, 1.2)	(0.2, 1.8)	(0.5, 1.1)
*P* value	0.47	0.36	0.27	0.19	0.33	0.17
EC (μg/m^3^) IQRs	0.5	0.4	0.4	0.3	0.2	0.1
Estimate	1.10	0.90	0.90	0.60	0.50	0.70
95% CI	(0.8, 1.6)	(0.7, 1.2)	(0.6, 1.6)	(0.4, 1)	(0.3, 0.8)	(0.4, 1.1)
*P* value	0.48	0.60	0.82	0.05	0.01	0.11
CO (ppm) IQRs	0.5	0.6	0.6	0.5	0.3	0.1
Estimate	0.90	0.80	0.80	0.70	0.70	0.70
95% CI	(0.7, 1.2)	(0.5, 1.2)	(0.5, 1.3)	(0.4, 1.2)	(0.3, 1.8)	(0.4, 1.1)
*P* value	0.51	0.25	0.40	0.17	0.47	0.14
PM_2.5_ (μg/m^3^) IQRs	11.9	14.7	16.5	13.7	9.8	3.6
Estimate	1.00	0.90	0.80	0.70	0.50	0.50
95% CI	(0.8, 1.2)	(0.7, 1.2)	(0.5, 1.2)	(0.4, 1.1)	(0.2, 1.3)	(0.2, 1)
*P* value	0.84	0.44	0.30	0.11	0.18	0.05

All results are multiplicative changes in CC16 (ng/mL) per interquartile range (IQR) of the pollutant. These estimates come from a GAM model that adjusted for whether or not the child lives with a smoker, whether or not the child is Latinx, physical activity, household income, and a smoothed term for day of study.

In sensitivity analyses, the pattern of findings was unchanged when CC16 was adjusted for creatinine. The pattern of findings was also robust to the choice of the smoothing function for seasonality.

## Discussion

In this well-characterized 9-year-old child cohort, we found a consistent pattern of decreased HDL cholesterol across all NO_*x*_ and NO_2_ exposure time frames and longer-term (1-, 3-month and 1-year) time frames for most pollutants, a pattern of elevated 8-isoprostane levels during short-term (1-day, 1-week) exposure periods, non-significant increases in percent HbA1c during 1-month exposure time frames, and non-significant decreases in CC16 during 3-month exposure time frames. These results indicate a relationship between TRAP and markers of oxidative stress and metabolism in 9-year-old children, as previously found in this cohort at age 7, and possible associations with lung epithelial injury as well.

HDLs play a role in cardiovascular protective actions by eliminating excess cholesterol in arterial walls and providing anti-inflammatory properties [31]. Decreasing HDL cholesterol with increasing levels of air pollution in this study is consistent with previous adult epidemiologic studies demonstrating increased metabolic dysregulation associated with higher air pollutant exposure [32–34]. Other studies in pediatric populations have demonstrated associations between exposure to particulate matter, NO_*x*_ or combined pollutant indices with worsened biomarkers of metabolic dysregulation (plasma insulin, fasting glucose, oxidized low-density lipoprotein), and oxidative stress (malondialdehyde), as well as anthropometric measures (elevated BMI, systolic and diastolic blood pressure) [19, 35]. However, the results in this study contrast those of a cross-sectional analysis conducted in an Italian birth cohort that found no association between TRAP and HDL cholesterol [36]. This may be attributable to differences in study population characteristics, as in the Italian cohort, 9.29% were obese and overweight, while the CHAPS cohort has 48.7% obese and overweight children. It is possible that children who are overweight could be more sensitive to the effects of air pollution on HDL, as has previously been shown for air pollution effects on pediatric blood pressure [37]. The relationship between TRAP and HDL cholesterol in pediatric populations may be somewhat variable based on underlying population characteristics and is therefore worthy of further study.

TRAP exposure has been found in both experimental and epidemiologic studies to increase levels of reactive oxygen species that lead to higher levels of oxidative stress, resulting in degradation of important cellular molecules, such as lipids including HDL cholesterol [8, 33, 38]. For instance, an experimental study using cultured pulmonary alveolar macrophages exposed to PAHs indicated that these compounds were metabolized by cytochrome P450A1 into quinones contributing to the generation of reactive oxygen species, thereby increasing levels of oxidative stress [39]. This mechanism aligns with our study’s results on urinary 8-isoprostane, a stable biomarker of lipid peroxidation, which was elevated in association with short-term exposure periods of most of the TRAP we studied, but the association dissipated for longer exposure averages. This is consistent with the known half-life of 8-isoprostane (roughly 16 min in serum, likely moderately longer in urine) [40]. Similarly, a large cross-sectional study of 2035 adult participants in Framingham, MA, found that 3- to 7-day moving averages of black carbon (BC) and NO_*x*_ exposure were also shown to be associated with increased urinary 8‐isoprostane [41]. Moreover, pediatric studies have also detected elevated concentrations of 8-isoprostane in exhaled breath condensates from children linked to BC exposure [42, 43]. The prior CHAPS analyses looking at the cohort at age 7 found that short-term average TRAP exposure (1-day, 1-week, and 1-month) was consistently and significantly associated with creatinine-adjusted urinary 8-isoprostane [23]. Of the four pollutants assessed in the prior analysis—EC, NO_2_, PAH456, and PM_2.5_—the findings for the first three were very similar to those of the current study approximately 2 years later, with more precision in the estimates in the larger cohort at age 7. Interestingly, there was not a clear association between PM_2.5_ exposure and increased 8-isoprostane in this analysis, whereas there had been at age 7. These findings provide further evidence that air pollution can lead to oxidative stress in children as well as adults. Cytokine release from the inflammatory response to oxidative stress-induced lung injury can spill over into the circulatory system to cause systemic inflammation [4, 12] and increase risk for several chronic disorders, including metabolic syndrome, atherosclerotic cardiovascular disease, and type II diabetes [8].

HbA1c in this study showed a pattern of elevations associated with the 1-month moving average across several pollutants, most notably PAH456 and PM_2.5_, which can be attributed to circulating red blood cells having a life span of approximately 3–4 months [44]. Because red blood cells constantly turn over, the 1-month average will have a higher percentage of assayed cells that were present during that entire exposure duration, and this exposure window will represent most of the exposure window for half or more of the red cells present. The prior CHAPS paper analyzing the 7-year-old cohort found significant associations between 3- and 6-month TRAP exposure and increased percent changes of HbA1c [23]. In adults, air pollution exposure is thought to contribute to type II diabetes [1, 45, 46]. A recent birth cohort study found that prenatal exposure to PM_2.5_ was associated with increased HbA1c levels in prepubertal children of ages 4–5 years, suggesting that this relationship could hold for recent exposure in older children as well [47]. Experimental animal studies observed exposure to particulate matter to be associated with changes in insulin sensitivity and amplified adipose inflammation in mouse models of diet-induced obesity [5], as well as induced in vivo expression of metabolic syndrome-related genes in mice, specifically genes related to inflammation, lipid and cholesterol metabolism, and atherosclerosis [48]. Based on this evidence from air pollution exposure in animal and epidemiology studies, insulin resistance, dyslipidemia, and central adiposity may be related to particulate matter exposure via inflammatory pathways. These findings contribute to the limited literature thus far assessing this pathway in children.

CC16, a biomarker of lung epithelial damage, is an anti-inflammatory protein secreted from club cells in response to oxidative stress and inflammation [49]. We found a consistent trend of decreasing CC16 with longer exposure periods (3 and 6 months) but with several confidence intervals crossing the null. Studies have shown that chronic exposure to air pollution, and especially to tobacco smoke, is associated with lower levels of CC16 and increased risk of chronic obstructive pulmonary disease [50], while short-term exposure is associated with elevated levels of CC16 [51]. Because our outcomes were measured at one time point and associated with multiple pollutant exposure windows, it may be that effects in opposite directions obscure the short-term findings. In a longitudinal birth cohort study following participants from age 6–32, higher levels of early-life exposure to NO_2_ were associated with consistently lower levels of circulating CC16, indicating that increased NO_2_ exposure during childhood may impact critical windows of lung development [49]. Because prior studies suggest that CC16 may be associated with long-term exposure to air pollution and decreases in lung function, it is particularly important that this relationship continue to be explored.

This research study has several strengths. These include a comprehensive and high-quality set of exposure data (including novel pollutants such as ambient PAHs), as well as a careful assessment of biomarkers. This study also adds to the literature on health effects of pollution for children of color from low-income families.

Limitations include the cross-sectional analysis and relatively small sample size. Due to the large number of pollutants and exposure time frames, we mitigated the risk of type II errors from multiple comparisons by interpreting the results based on the general patterns of confidence intervals rather than looking at the significance level of each statistical test. A complete lipid panel for the study would have been preferable, but we tested only for HDL cholesterol instead since it does not require fasting prior to collection, creating less burden on participants.

Future avenues of research include running longitudinal analyses of the data from the CHAPS cohort at both ages 7 and 9. Longitudinal analyses were not practical for some of these outcomes as most of the cohort did not have HDL cholesterol and none had CC16 measurements at age 7. Longitudinal assessments of the biomarkers and anthropometric data available at both time points are forthcoming in a future analysis.

Overall, our results support the hypothesis that acute exposure to TRAP impacts metabolic function in children. Low-grade systemic inflammation is associated with metabolic syndrome in adults, and is an important factor in instigating premature atherosclerosis [52]. For this reason, it is crucial to consider whether early-life exposure to ambient air pollution could contribute to later-life cardiometabolic disease [17, 53]. Evidence of linkage between TRAP exposure and the biomarkers measured in our study suggests that air pollution contributes to abnormal lipid and glucose metabolism in children, which may then lead to increased risk of metabolic syndrome in adulthood. This relationship between TRAP and metabolic function in children argues for public health actions that could further decrease exposures to air pollution during childhood.

## Supplementary information

Supplementary information

Supplementary information

Supplementary information

Supplementary information

Supplementary information
