# Supplementary material for: Traffic-related air pollution, biomarkers of metabolic dysfunction, oxidative stress, and CC16 in children
Source: J Expo Sci Environ Epidemiol. 2021 Aug 20;32(4):530–7. doi: 10.1038/s41370-021-00378-6 (PMC8858324; doi:10.1038/s41370-021-00378-6)
Supplement: Supplementary file 1 — Supplementary information [file 41370_2021_378_MOESM1_ESM.docx]

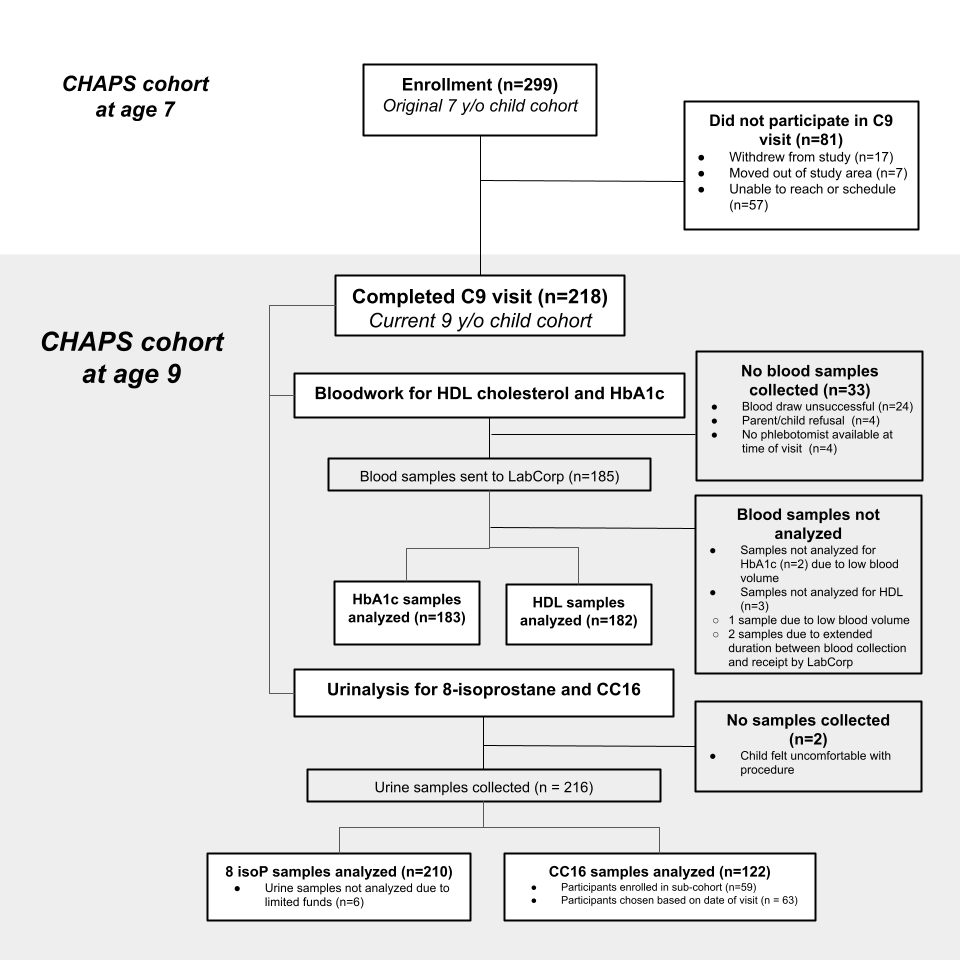
**Supplemental Figure 1: Participant workflow diagram.** From the 7-year-old child cohort from a previous study, this figure illustrates the number of participants that were retained for this 9-year-old child cohort study, as well as the number with bloodwork done for HDL cholesterol and urinalysis for 8-isoprostane (23).
