# Supplementary material for: Traffic-related air pollution, biomarkers of metabolic dysfunction, oxidative stress, and CC16 in children
Source: J Expo Sci Environ Epidemiol. 2021 Aug 20;32(4):530–7. doi: 10.1038/s41370-021-00378-6 (PMC8858324; doi:10.1038/s41370-021-00378-6)

**Supplemental Fig 2: DAG (directed acyclic graph)**. Covariates variables to include in the regression model were determined using a directed acyclic graph.


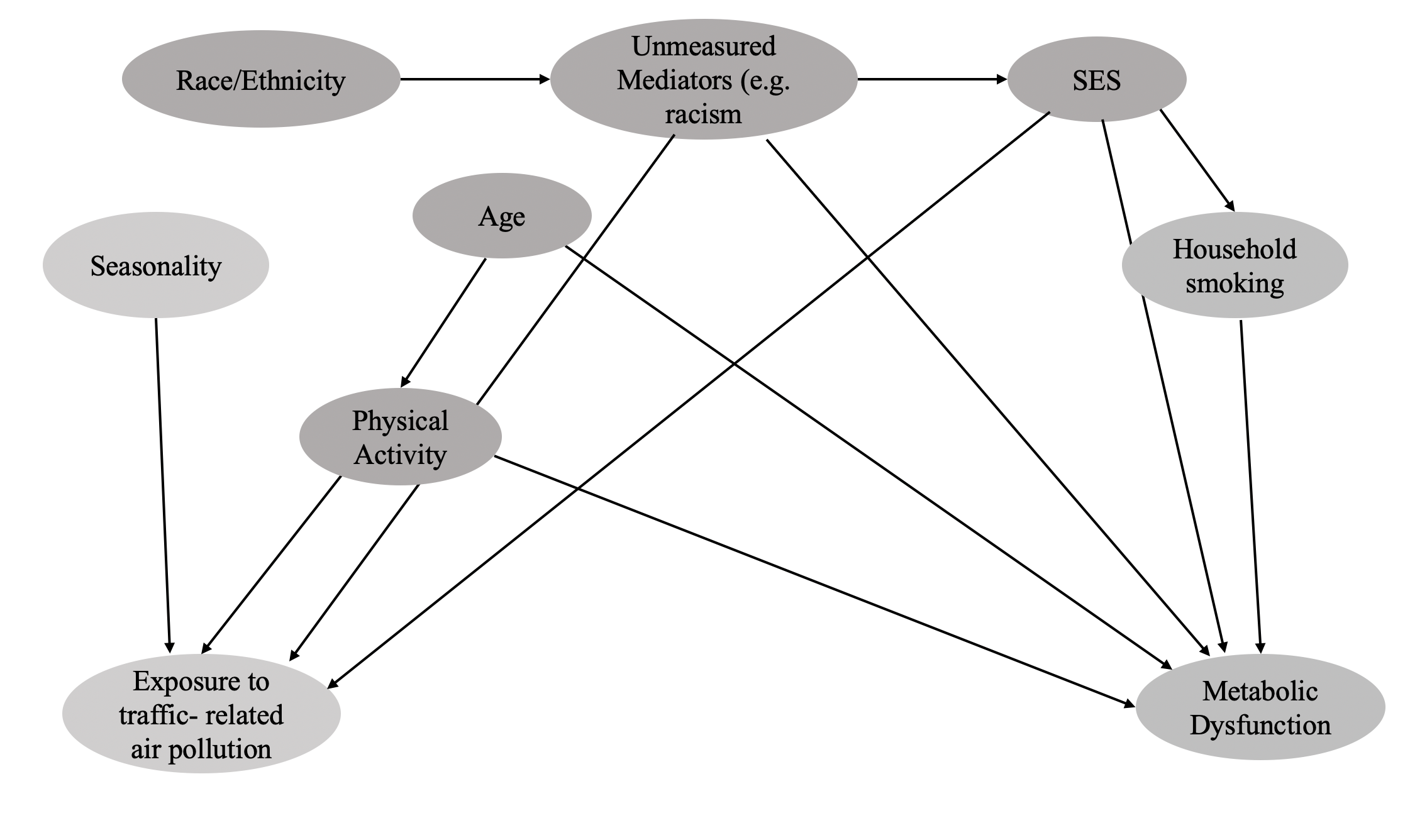

Supplement: Supplementary file 2 — Supplementary information [file 41370_2021_378_MOESM2_ESM.docx]
