# Supplementary material for: Traffic-related air pollution, biomarkers of metabolic dysfunction, oxidative stress, and CC16 in children
Source: J Expo Sci Environ Epidemiol. 2021 Aug 20;32(4):530–7. doi: 10.1038/s41370-021-00378-6 (PMC8858324; doi:10.1038/s41370-021-00378-6)
Supplement: Supplementary file 3 — Supplementary information [file 41370_2021_378_MOESM3_ESM.docx]

| **Pollutant** | **1-day average** | **1-week average** | **1-month average** | **3-month average** | **6-month average** | **1-year average** |
| --- | --- | --- | --- | --- | --- | --- |
| NO_2_ (ppb) IQRs | 7.31 | 7.92 | 8.98 | 8.48 | 8.72 | 8.30 |
| 25th %le | 4.12 | 4.53 | 4.33 | 4.46 | 5.64 | 7.23 |
| 75th %le | 13.54 | 14.58 | 14.05 | 13.75 | 11.65 | 9.40 |
| NO_X_ (ppb) IQRs | 14.59 | 17.06 | 16.59 | 16.00 | 16.38 | 17.15 |
| 25th %le | 10.06 | 10.53 | 10.79 | 11.47 | 13.00 | 15.36 |
| 75th %le | 23.16 | 23.89 | 25.48 | 24.03 | 21.69 | 18.84 |
| PAH456 (ng/m^3^) IQRs | 10.22 | 10.84 | 10.47 | 9.72 | 9.50 | 9.47 |
| 25th %le | 5.84 | 5.76 | 5.55 | 6.11 | 6.94 | 9.11 |
| 75th %le | 13.50 | 13.70 | 13.95 | 13.99 | 12.18 | 9.90 |
| EC (μg /m^3^) IQRs | 0.50 | 0.56 | 0.56 | 0.61 | 0.62 | 0.60 |
| 25th %le | 0.29 | 0.37 | 0.38 | 0.41 | 0.51 | 0.55 |
| 75th %le | 0.82 | 0.76 | 0.76 | 0.75 | 0.75 | 0.66 |
| CO (ppm) IQRs | 0.37 | 0.47 | 0.49 | 0.48 | 0.47 | 0.48 |
| 25th %le | 0.20 | 0.21 | 0.23 | 0.26 | 0.35 | 0.45 |
| 75th %le | 0.73 | 0.81 | 0.80 | 0.75 | 0.63 | 0.50 |
| PM_2.5_ (μg /m^3^) IQRs | 11.10 | 11.57 | 13.58 | 15.31 | 16.43 | 15.35 |
| 25th %le | 7.42 | 8.15 | 7.89 | 7.64 | 10.29 | 13.08 |
| 75th %le | 19.27 | 22.88 | 24.37 | 21.37 | 20.13 | 16.70 |

**Supplemental Table 1:** Summary characteristics of air pollution exposure data. Median, 25th percentile, 75th percentile for pollutant exposures (NO_2_, NO_X_, PAH456, EC, CO, PM_2.5_)
