# Supplementary material for: Traffic-related air pollution, biomarkers of metabolic dysfunction, oxidative stress, and CC16 in children
Source: J Expo Sci Environ Epidemiol. 2021 Aug 20;32(4):530–7. doi: 10.1038/s41370-021-00378-6 (PMC8858324; doi:10.1038/s41370-021-00378-6)
Supplement: Supplementary file 4 — Supplementary information [file 41370_2021_378_MOESM4_ESM.docx]

**Supplemental Table 2.** Descriptive statistics for biomarkers of metabolic dysregulation, oxidative stress and lung epithelial damage.

| **Biomarker** | **N** | **Mean [SD]** | **Median (Q1, Q3)** |  |
| --- | --- | --- | --- | --- |
| High density lipoprotein (mg/dL) | 182 | 51.29 [12.16] | 50.0 (42.00, 60.00) |  |
| 8-isoprostane : Creatinine ratio (ng/mg)^a^ | 210 | 6.20 [8.01] | 4.52 (2.62, 7.57) |  |
| Club cell protein 16  (ng/mL)^a^ | 122 | 4.01 [6.55] | 1.97 (0.75, 4.32) |  |
| Glycosylated hemoglobin A1c (%) | 183 | 5.26 [0.30] | 5.30 (5.10, 5.40) |  |
|  |  |  |  |  |
| ^a^ 8-isoprostane and CC16 were not normally distributed and were logged for analysis. | | | | |
