# Supplementary material for: Traffic-related air pollution, biomarkers of metabolic dysfunction, oxidative stress, and CC16 in children
Source: J Expo Sci Environ Epidemiol. 2021 Aug 20;32(4):530–7. doi: 10.1038/s41370-021-00378-6 (PMC8858324; doi:10.1038/s41370-021-00378-6)
Supplement: Supplementary file 5 — Supplementary information [file 41370_2021_378_MOESM5_ESM.docx]

| Supplemental Table 3a: Outcomes Correlation Matrix | | | |
| --- | --- | --- | --- |
|  |  |  |  |
|  | **8-isoprostane** | **CC16** | **HbA1c** |
| **HDL** | -0.06 | 0.15 | 0.07 |
| **8-isoprostane** |  | 0.03 | -0.05 |
| **CC16** |  |  | 0.12 |

| Supplemental Table 3b: Exposure Correlations by Pollutant and Exposure Duration | | | | | |
| --- | --- | --- | --- | --- | --- |
|  |  |  |  |  |  |
|  | **1-Day Lag** | | | | |
|  | **PM_2.5_** | **NO_2_** | **NO_X_** | **PAH456** | **EC** |
| **CO** | 0.79 | 0.85 | 0.81 | 0.81 | 0.58 |
| **PM_2.5_** |  | 0.83 | 0.71 | 0.67 | 0.63 |
| **NO_2_** |  |  | 0.89 | 0.83 | 0.72 |
| **NO_X_** |  |  |  | 0.83 | 0.64 |
| **PAH456** |  |  |  |  | 0.50 |
|  |  |  |  |  |  |
|  | **1-Month Average** | | | | |
|  | **PM_2.5_** | **NO_2_** | **NO_X_** | **PAH456** | **EC** |
| **CO** | 0.91 | 0.96 | 0.93 | 0.95 | 0.67 |
| **PM_2.5_** |  | 0.91 | 0.89 | 0.90 | 0.74 |
| **NO_2_** |  |  | 0.97 | 0.92 | 0.77 |
| **NO_X_** |  |  |  | 0.92 | 0.70 |
| **PAH456** |  |  |  |  | 0.60 |
|  |  |  |  |  |  |
|  | **6-Month Average** | | | | |
|  | **PM_2.5_** | **NO_2_** | **NO_X_** | **PAH456** | **EC** |
| **CO** | 0.94 | 0.95 | 0.88 | 0.98 | 0.66 |
| **PM_2.5_** |  | 0.92 | 0.83 | 0.90 | 0.76 |
| **NO_2_** |  |  | 0.97 | 0.93 | 0.71 |
| **NO_X_** |  |  |  | 0.87 | 0.61 |
| **PAH456** |  |  |  |  | 0.60 |

|  | **1-Week Average** | | | | |
| --- | --- | --- | --- | --- | --- |
|  | **PM_2.5_** | **NO_2_** | **NO_X_** | **PAH456** | **EC** |
| **CO** | 0.90 | 0.96 | 0.92 | 0.91 | 0.72 |
| **PM_2.5_** |  | 0.93 | 0.90 | 0.84 | 0.77 |
| **NO_2_** |  |  | 0.97 | 0.89 | 0.81 |
| **NO_X_** |  |  |  | 0.91 | 0.74 |
| **PAH456** |  |  |  |  | 0.58 |
|  |  |  |  |  |  |
|  | **3-Month Average** | | | | |
|  | **PM_2.5_** | **NO_2_** | **NO_X_** | **PAH456** | **EC** |
| **CO** | 0.93 | 0.97 | 0.93 | 0.98 | 0.67 |
| **PM_2.5_** |  | 0.93 | 0.90 | 0.91 | 0.78 |
| **NO_2_** |  |  | 0.98 | 0.94 | 0.73 |
| **NO_X_** |  |  |  | 0.92 | 0.67 |
| **PAH456** |  |  |  |  | 0.61 |
|  |  |  |  |  |  |
|  | **1-Year Average** | | | | |
|  | **PM_2.5_** | **NO_2_** | **NO_X_** | **PAH456** | **EC** |
| **CO** | 0.83 | 0.79 | 0.52 | 0.63 | 0.66 |
| **PM_2.5_** |  | 0.72 | 0.39 | 0.57 | 0.55 |
| **NO_2_** |  |  | 0.88 | 0.66 | 0.69 |
| **NO_X_** |  |  |  | 0.47 | 0.55 |
| **PAH456** |  |  |  |  | 0.68 |
